# The role of worldviews, radicalization risk factors, and personality in harassment of scientists

**DOI:** 10.1038/s41598-025-85208-7

**Published:** 2025-01-08

**Authors:** Vukašin Gligorić, Carlotta Reinhardt, Ella Nieuwenhuijzen, Josha Orobio de Castro, Allard R. Feddes, Gerben A. van Kleef, Bastiaan T. Rutjens

**Affiliations:** 1 Department of Psychology, University of Amsterdam, Nieuwe Achtergracht 129B, 1018 WS Amsterdam, The Netherlands; 2 University of Hamburg, Hamburg, Germany

**Keywords:** Scientist harassment, Violence toward scientists, Science attitudes, Perception of scientists, Radicalization, Psychology, Psychology and behaviour

## Abstract

Anti-science movements brought more than public distrust in science. Perhaps even more worryingly, these movements are also associated with instances of harassment of—and violence against—scientists. However, virtually nothing is known about individuals likely to harass or harm scientists. Across two pre-registered studies (total N = 749), we investigated the role of worldviews (e.g., political ideology, conspiracy mentality, science cynicism), radicalization risk factors (relative deprivation and threat), and personality traits and how these relate to harassment of scientists (both attitudes and behavior). We found that science cynicism–the perception that scientists are incompetent and corrupt–drives approval of scientists’ harassment (attitudes), as well as harmful behavior (e.g., refusing to donate money, not signing a petition). Additionally, perceiving scientists as threatening, as well as dark personality traits (psychopathy and narcissism), contributed to approving scientists’ harassment. Overall, the present research takes a first step in identifying predictors of the willingness to harm scientists.

COVID-19 did not impact only (physical) health: in a dramatic case during the pandemic, a Dutch virologist and a prominent member of the governmental COVID-19 management team barely escaped an angry mob when she walked into a museum in Amsterdam. Although seemingly an extreme example, her case is not an exception: as much as 38% of scientists who worked on pandemic-related research faced different forms of harassment (from personal insults and doxing to wishes of harm or death, O’Grady^1^), while 15% received death threats^2^ (note that respondents to these surveys were predominantly Western scientists). Given that a large number of scientists faced such extreme and harmful behavior—and even suffered physical attacks^2^—it is clearly important to explore the factors driving it. However, to our knowledge, no research so far has done so. The present work aims to fill this gap by examining which individuals are more likely to engage in harassing scientists. Drawing on previous research on science attitudes which showed that various worldviews (e.g., religiosity, conservatism) are key predictors of negative attitudes toward science and scientists^3–5^, we investigate the role of the following worldviews: religiosity, spirituality, political ideology, conspiracy mentality, science populism, and science cynicism. Additionally, we draw on the radicalization literature^6^ and investigate the role of relative deprivation and threat as radicalization risk factors. In Study 2, we extend this approach by including personality factors, specifically the dark triad (narcissism, Machiavellianism, and psychopathy). Besides measuring attitudes toward scientist harassment, we also include behavioral measures of aggression and violence toward scientists. In the next sections, we provide a brief background to each of these factors.

## Worldviews shape negative science attitudes

Even though research has not specifically investigated which individuals are prone to violence against scientists, examining general attitudes toward science might unveil potential factors influencing such tendencies. Particularly prevalent since the COVID-19 pandemic, anti-science attitudes and skepticism towards science have been increasing over the past decades^7–10^. Mistrust in science, and ultimately the dismissal of scientific evidence, can occur when science does not align with a person’s worldview or ideologies^3^. One such worldview could be religion, as it offers competing explanations for many phenomena (e.g., the origin of Earth and life, evolution, afterlife^11,12^). Indeed, research has found that believing in God, as well as higher religiosity overall, are associated with more negative attitudes to, and less trust in, science and scientists (e.g.,^13–15^). Besides religion, spirituality is another driver of negative science attitudes, as it has been shown to predict lower faith in science and vaccination skepticism (e.g.,^16^). Although there is a relation between spirituality and religiosity (many religious people will also identify as spiritual), research generally focuses on post-Christian spirituality and so-called spiritual-but-not-religious believers (e.g.,^17^). Therefore, we treat these two worldviews separately.

Not only religiosity and spirituality are related to distrust in science and scientists. Conservative ideology seems to be in conflict with (at least some) science, given that political conservatives show lower faith in science^18^, increasing distrust^8^, and more anti-science attitudes^19^. Additionally, most scientists are liberal-leaning and as such perceived by some to push liberal values in science^20^. Conservatives’ distrust particularly translates to specific areas of science, most prominently climate change^21^ or COVID-19^22,23^. Recently, Gligorić and colleagues^24^ found that, in the US, conservatives (vs liberals) showed lower trust in 43 out of 45 studied scientific occupations (e.g., biologists, climatologists, sociologists). Overall, research suggests that political conservatives have more negative attitudes toward science and scientists.

While most science attitude research has focused on religious and spiritual belief, science knowledge, and political ideology^25^, recent research has started to investigate the influence of distrusting worldviews, which are not necessarily related to religious or political ideology. Most notably, COVID-19 brought an exponential increase in conspiracy theories. For example, various studies found that the prevalence of most COVID-19 conspiracy theories (e.g., the virus originated from a laboratory or was created by big pharmaceutical companies, or that it was made to produce destabilization) is over 20%^26^. These explanations challenge the official and evidence-based ones, questioning the origin of the virus, its prevention, and treatment. Thus, it is not surprising that belief in conspiracy theories is one of the most important drivers of refusing to engage in COVID-19 protective behaviors^27^, and one of the strongest predictors of skepticism toward COVID-19 vaccines^28^, as well as childhood vaccines^29^. More generally, conspiracy beliefs about how those in power (including scientists) have malevolent goals contribute to science rejection^4,30^.

Mistrust in scientists does not necessarily have to come in the form of conspiracy theories, i.e., narratives challenging official explanations. Populism–a view that distinguishes between corrupt elite groups (i.e., establishment) and good-hearted ordinary people–could also be a contributing factor, given that scientists can be seen as a part of the establishment (e.g.,^31^). Specific to science, researchers developed the concept of “science-related populism” as a distinct variant of populism. In the view of science populists, a powerful elite of academics stands against the ordinary people,the elite decides (in a biased way) what will be studied and funded (research agenda), as well as what constitutes the truth (knowledge). On the contrary, what ordinary people consider to be important and true (using common sense) is excluded from this decision-making^32,33^. Indeed, individuals who hold science-populist attitudes have more negative attitudes toward science and scientists (e.g., lower trust in science, scientists, science coverage, and impact of science^33^, also see^34^). Interestingly, populism and conspiratorial worldview share dispositional distrust as their central characteristic^35^, and are both related to cynicism^36^—a view that others such as groups and institutions are immoral, unreliable, and corrupt. In general, cynicism views human nature as inherently bad and motivated by self-interests^37^, which could also impact the perception of scientists. In sum, conspiracy mentality, science-populism, and science cynicism center around distrust of institutions and academics and thus shape negative attitudes toward science.

Although the worldviews above relate to negative attitudes toward science and scientists, attitudes alone would not likely lead to harassment of scientists, which ranges from insults to vandalism and death threats^2,37^. However, besides relating to negative attitudes toward science, some of the worldviews above also relate to violence. Notably, right-wing ideology and conservatism are associated with violence-favoring attitudes and aggressive behavior^38,39^. Similarly, populism^40^ and cynicism^41^ have been found to be associated with violence. Conspiracy mentality is another well-established driver of violence, as it was found to predict support and intention to engage in violence^42–44^. In sum, the worldviews above have been associated with both negative attitudes toward science/scientists and a (general) propensity to violence, making them potential drivers of scientists’ harassment. Importantly, given that harassment of scientists can be seen as an example of radical and extremist behavior, we next turn to literature on radicalization.

## Risk factors of radicalization: relative deprivation and threat perception

Radicalization is defined as a process through which people become increasingly motivated to use violent means against members of an out-group or symbolic targets to achieve behavioral change and political goals^6^. Radicalization frameworks could be useful to understand why some individuals would harass scientists, although there are noteworthy differences between the prototypical radicalization of political and religious groups and anti-science movements (e.g., absence of clear ingroup identity or coherent ideology). Since radicalization is a complex and multi-faceted issue, we selected those drivers of radicalization that are pertinent in the case of scientists’ harassment. Thus, we focused on *risk factors*, as one of the three different categories as root drivers of radicalization (other being trigger factors and psychological needs^45^). Risk factors represent contextual forces or states that make individuals more prone to radicalization (compared to trigger factors which are discrete events, e.g., the death of a family member^45,46^).

Wolfowicz and colleagues^47^ identified as many as 52 risk factors for radical attitudes, noting that factors regarding national and/or religious identity, and collective relative deprivation were found to be most commonly identified as predictors of radicalization. Relative deprivation (which can be felt at the individual or collective level) refers to perceiving or feeling treated less well (as an individual or as a group) than what is considered just and deserved. In the present research, we focused on relative deprivation (individual and collective^48^) considering that people may feel deprived as a consequence of the behavior of an out-group (scientists).

Apart from relative deprivation, we identified threat perception—one of the strongest risk factors that affect radicalization^47,48^—as a potential driver of aggressive tendencies toward scientists. These threat perceptions can be realistic (relating to resources or physical safety) or symbolic (relating to values^49^). Indeed, many scientific areas can be seen as threatening (e.g., nuclear energy, climate change), both physically (possibilities of injuries or death) and symbolically (e.g., less freedom to respond to dangers). This was, for example, the case with the COVID-19 outbreak which was perceived as a realistic and symbolic threat^50^. However, in the case of harassment of scientists, it is not the societal problem (e.g., virus outbreak, climate change) that is perceived as threatening, but scientists’ proposed solutions (e.g., vaccination, regulation of economy). Therefore, science and scientists can be seen as threatening one’s physical and economic safety, or values such as freedom^51,52^. Such threat perception could then fuel radical behavior toward scientists such as harassment.

## The role of personality in negative and violent attitudes

Besides worldviews and radicalization risk factors, personality factors play an important role in extremely negative and violent attitudes. In particular, the dark triad—which refers to a constellation of three interrelated negative personality traits: (subclinical) narcissism, Machiavellianism, and (subclinical) psychopathy—has been associated with negative and violent attitudes^53,54^. The trait of narcissism is characterized by pursuing gratification from egotistical admiration of the individual’s attributes. Machiavellianism refers to a deceitful and manipulative trait that entails the focus on self-interest with no regard for others. Psychopathy is characterized by anti-social behavior, a lack of empathy and remorse, and impulsiveness. The core of these three traits (which was also proposed to reflect the term “evil”^55^) seems to be low agreeableness^56^, and manipulation and callousness^57^. Therefore, it is no surprise that a strong body of research showed that all three traits predict aggression and violence^53,56,58,59^.

## The present research

In the present research, we aimed to identify predictors of scientists’ harassment by focusing on three groups of predictors—worldviews (religiosity, spirituality, political ideology, science populism, science cynicism, and conspiracy mentality; Studies 1 and 2), risk factors for radicalization (individual and collective relative deprivation, symbolic and realistic threat; Studies 1 and 2), and the three dark triad personalities (narcissism, Machiavellianism, and psychopathy; Study 2). To measure scientists’ harassment, we included attitudes toward different forms of harassment of scientists (e.g., insults, death threats; Studies 1 and 2), and three different behavioral variables. In Study 1, we used the Voodoo Doll Task^60^ in which participants expressed their aggression by clicking on a doll representing a stereotypical scientist. In Study 2, we asked participants: (1) to donate money to the cause of protecting scientists against harassment and (2) to sign an online petition with the same goal. Materials, data, and analysis code are available at the Open Science Framework (https://osf.io/7rzce/?view_only=957180de7bcc4d0c88dedc2219e52816). Preregistration for Study 1 is available on https://osf.io/q2x67/?view_only=88f68b5093d04dbc8d9e9e0f249559d2, while preregistration for Study 2 can be found on https://osf.io/gcu6h/?view_only=6b1a346e88b94579a8cc7ce1475a49c9. We note that although the study was pre-registered, we did not have any specific hypotheses but wanted to explore potential predictors.

## Study 1

### Methods

#### Participants

Given that we were interested in examining the pattern of correlations between multiple predictors and outcomes (rather than testing a specific hypothesis), we focused on the smallest correlation coefficient of interest for power analysis calculation. We set the minimum sample size at 250, which is needed for correlations of *r* = 0.20 to stabilize^61^. However, we aimed to collect as many participants as possible to get more precise estimates. In total, 457 participants were recruited through social networks such as Instagram, Reddit, and Facebook to complete the study and earn the possibility to win a 15 euro gift card in return. After excluding 18 speeders (completion time at least twice as fast as the median) and 26 outliers (six multivariate on all independent and dependent variables and 20 univariate on behavioral dependent variable), we were left with the total sample of *N* = 413 (281 males, *M*_age_ = 35.5). The majority of the participants were from either the United States (45.5%) or the Netherlands (37.3%; distribution of participants’ countries is given in the supplement at OSF). Regarding education, 1.2% indicated not having completed high/secondary school, 18.9% had completed high/secondary school, 15% were currently studying, 38.3% had an undergraduate degree and 26.6% had a graduate degree.

#### Materials and procedure

After reading the information letter and giving their consent, participants reported socio-demographic characteristics (country, sex, age, education). Next, participants completed the following scales in the order presented here (see the OSF for the complete questionnaire). All materials were presented in English.

##### Political ideology

Political ideology was measured with two items: participants indicated their ideology on social issues (e.g., minority rights, gender equality) and economic issues (planned vs. free-market economy) respectively on a scale from *left-wing* (1) to *right-wing* (9)^62^. The two items were “Please indicate your ideological stance on social issues (e.g. minority rights, gender equality). People on the left tend to change the traditional and cultural norms, while people on the right tend to preserve them.” and “Please indicate your ideological stance on economic issues (planned vs. free-market economy). People on the left prefer state/public ownership and equal wealth distribution, while people on the right prefer private ownership and individual responsibility.” Items correlated strongly, *r* = 0.67.

##### Religiosity

Religiosity was measured using the Centrality of Religiosity Scale^63^ which contains 5 items such as “How often do you think about religious issues?” and “To what extent do you believe that God or something divine exists?”. Items were rated on a 5-point Likert scale ranging from *never* (1) to *very often* (5), or *not at all* (1) to *very much so* (5). The scale showed good reliability (α = 0.88).

##### Spirituality

Spirituality was measured using two items: participants indicated what extent they consider themselves to be a spiritual person and to what extent other individuals consider them to be a spiritual person on a scale from *not at all* (1) to *completely* (7)^64^. Items showed a high correlation, *r* = 0.80.

##### Conspiracy mentality

Conspiracy mentality was measured using the six (The original scale has 7 items, however, due to a programming mistake we did not include the seventh item in the scale (which had the lowest factor loading in the original subscale: “Events on the news may not have actually happened”). This applies to both studies.) items from the subscale *Conspiracy Theory Ideation* (CTI) that contained items such as “The government or covert organizations are responsible for events that are unusual or unexplained” and “Many so called “coincidences” are in fact clues as to how things really happened”^65^. Items were rated using a 7-point Likert scale ranging from *strongly disagree* (1) to *strongly agree* (7). The scale had a good reliability (α = 0.88).

##### Science populism

Science populism was measured using six items (e.g., “We should rely more on common sense and less on scientific studies”; “People like me should be involved in decisions about the topics scientists research”) of the SciPop Scale rated on a 5-point Likert scale ranging from *fully disagree* (1) to *fully agree* (5)^33^. The scale showed acceptable reliability (α = 0.76).

##### Science cynicism

Science cynicism was measured using four items of the Political Cynicism Scale^66^ which was adapted to “scientists” (instead of “politicians”). The scale included items such as “It is easier to become a scientist thanks to academic friends than because of competence”, “Scientists are mainly focused on their own interests”, and “Scientists do not understand what is happening in society”. Ratings were given on a 5-point Likert scale ranging from *fully disagree* (1) to *fully agree* (5). The scale showed good reliability (α = 0.81).

##### Relative deprivation

To measure individual relative deprivation, participants rated their agreement with six items (e.g., “I don’t think I get as many chances as others in [country]”; “It makes me angry when I think of how I am treated compared to others”^48^). Collective relative deprivation was measured in a similar way using an adapted six-item scale (“If I compare people like me with other groups in [country], I have the feeling that we are being treated unfairly”; “I believe people like me are discriminated against in [country]”)^48^. All items were rated on a 5-point Likert scale ranging from *totally disagree* (1) to *totally agree* (5). Both scales showed good reliability (both αs = 0.86). However, given the very high intercorrelation between the two scales (*r* = 0.87), we calculated one score of relative deprivation (α = 0.93), and further used it as one scale.

##### Threat

To measure symbolic threat, participants were asked to indicate the extent to which scientists are a threat to five symbolic issues (e.g., “values and traditions of [country]”, and “the rights and freedoms of the population of [country]”). Similarly, realistic threat was measured by asking about the perception of scientists as a threat to another five issues (e.g., to “personal health”, to “personal financial stability”^50^). Items were answered on a 4-point scale ranging from *not a threat* (1) to *major threat* (4). Both scales showed high reliability (respectively α = 0.91 and α = 0.92). Given the very high intercorrelation between the two scales (*r* = 0.84), we calculated one score of threat (α = 0.95), and treated it as one construct. Factor analyses for both Relative deprivation and Threat in both studies are available as supplemental information on OSF.

##### Attitudes towards scientist harassment

To measure harassment attitudes, we presented participants with 10 behaviors of scientists’ harassment such as insults, physical threats or intimidation, online intimidation, or death threats^37^, and asked participants to indicate whether they disapprove or approve of these behaviors. Items were rated on a 5-point Likert scale ranging from *total disapproval* (1) to *total approval* (5). The scale showed good reliability (α = 0.85).

##### Voodoo doll task

**Fig. 1:**
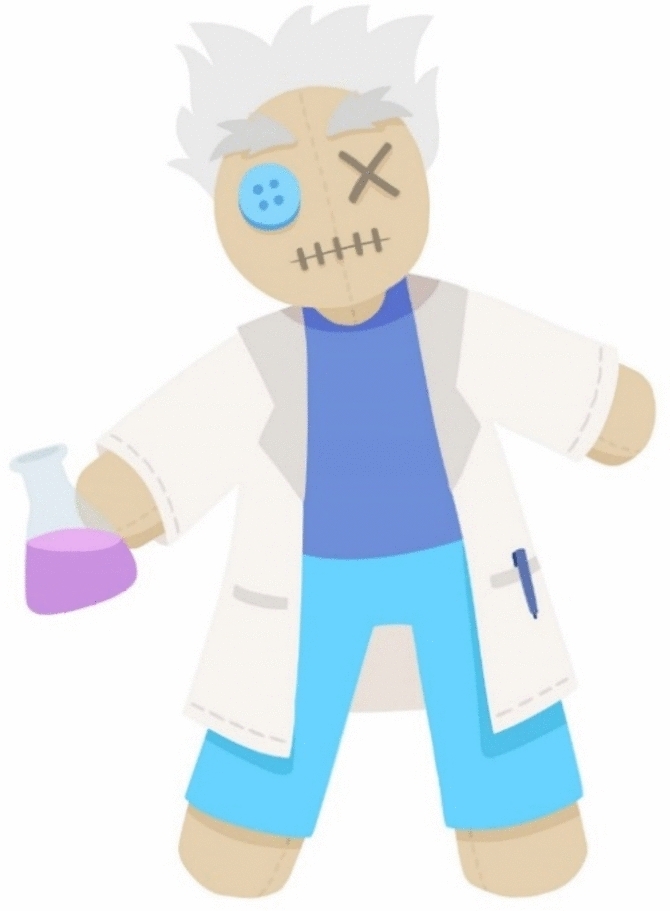
Voodoo doll scientists to which participants inserted pins by clicking on it.

Finally, to measure aggressive behavior toward scientists, we adapted the Voodoo Doll Task (validated previously in an online setting^60^). This task includes the presentation of a voodoo doll as a target on which participants can click to “release negative energy”. We developed the scientist voodoo doll inspired by the image of a stereotypical scientist^67^ which included an old-age male with a lab coat and equipment (Fig. 1). Participants were given the following instructions: “Because some aspects of this study can be experienced as negative, you now have the possibility to release this negative energy. To do so, you can insert pins into the scientist voodoo doll by clicking on it. Each click represents a pin. You can insert any number of pins that you want (even zero). Once you are done, please click the continue button.” A higher number of pins (clicks) indicated more aggressive behavior.

### Results

Table 1 contains means, standard deviations, and intercorrelations of variables (other descriptives are available on OSF as supplemental information). The correlations indicate that attitudes toward harassment were related to all worldviews and radicalization factors: right-wing ideology, religiosity, spirituality, conspiracy mentality, science populism and cynicism, feelings of relative deprivation and threat were all related to more approving attitudes toward harassment of scientists. Notably, these correlations varied substantially, from *r* = 0.25 to 0.64 (spirituality and threat perception respectively). The behavioral measure of aggressive behavior (voodoo pins) had significant positive correlations with five variables (conspiracy mentality, science cynicism, relative deprivation, threat, attitudes toward harassment), but these were noticeably lower (*r*s ranging from 0.11 to 0.18). Interestingly, it showed a relatively low correlation with attitudes (*r* = 0.11), indicating low convergent validity.

**Table 1:** Means, standard deviations and intercorrelations between variables.

	$M$	$SD$	1	2	3	4	5	6	7	8	9
1 Political ideology	4.03	2.07									
2 Religiosity	1.99	1.00	0.28***								
3 Spirituality	2.63	1.67	0.18***	0.73***							
4 Conspiracy mentality	3.26	1.39	0.33***	0.34***	0.34***						
5 Science populism	2.11	0.82	0.46***	0.29***	0.27***	0.59***					
6 Science cynicism	2.40	0.93	0.54***	0.30***	0.27***	0.56***	0.68***				
7 Relative deprivation	2.25	0.89	0.13**	0.19***	0.15**	0.37***	0.35***	0.35***			
8 Threat	1.42	0.69	0.50***	0.30***	0.27***	0.58***	0.64***	0.64***	0.31***		
9 Scientist harassment	1.41	0.49	0.39***	0.28***	0.25***	0.55***	0.59***	0.60***	0.31***	0.64***	
10 Voodoo pins	3.09	3.75	0.05	0.09	0.07	0.18***	0.09	0.15**	0.11*	0.17***	0.11*

*Note.* Correlations between predictor and outcome variables. * indicates *p* < 0.05, ** *p* < 0.01, *** *p* < 0.001.

Next, we wanted to assess the predictors’ relative importance in attitudes toward scientists’ harassment and aggressive behavior (voodoo pins) by conducting two multiple linear regression analyses. We report assumptions in the supplementary materials. We entered worldviews and radicalization factors as predictors. The results are provided in Table 2. For both dependent variables, the models were significant. Regarding attitudes toward scientists’ harassment, perception of threat arose as the strongest predictor, followed by distrusting worldviews (conspiracy mentality, science populism and science cynicism). As for the aggressive behavior (voodoo pins), even though the whole model was significant, no individual predictors were significant (also note the small *R*^2^). We also tested the robustness of the findings by running the same regression analyses while including age, education, and gender as a control. The pattern of results remained unchanged. These analyses are fully reported in the supplement on OSF. Finally, although we pre-registered the mediation analysis (attitudes toward harassment as a mediator of the predictors’ effect on voodoo doll pins), we did not run this analysis given that none of the predictors emerged as significant in predicting the voodoo doll pins.

**Table 2:** Worldviews and risk factors predicting attitudes toward scientist harassment and Voodoo Pin Task.

Model (DV and fit)	Scientist harassment					Voodoo pins				
	*F*(8,404) = 51.97, *p* < 0.001; *R*^2^ = 0.507 (adjusted *R*^2^ = 0.497)					*F*(8,404) = 2.74, *p* = 0.006; *R*^2^ = 0.052 (adjusted *R*^2^ = 0.033)				
Predictor	*β*	95%-CI	$t$	*p*	*VIF*	*β*	95%-CI	$t$	*p*	*VIF*
Political Ideology	0.00	[− 0.08, 0.09]	0.06	0.951	1.561	− 0.06	[− 0.18, 0.06]	− 0.96	0.339	1.561
Religiosity	0.04	[− 0.06, 0.15]	0.82	0.410	2.296	0.06	[− 0.09, 0.20]	0.78	0.437	2.296
Spirituality	-0.02	[− 0.12, 0.08]	− 0.42	0.677	2.229	− 0.04	[− 0.18, 0.11]	− 0.51	0.611	2.229
Conspiracy Mentality	**0.16**	**[0.06, 0.25]**	**3.28**	**0.001**	**1.892**	0.13	[− 0.00, 0.26]	1.96	0.050	1.892
Science Populism	**0.14**	**[0.04, 0.25]**	**2.71**	**0.007**	**2.338**	− 0.12	[− 0.27, 0.02]	− 1.67	0.095	2.338
Science Cynicism	**0.19**	**[0.08, 0.30]**	**3.47**	**0.001**	**2.446**	0.08	[− 0.06, 0.23]	1.13	0.260	2.446
Relative Deprivation	0.03	[− 0.05, 0.11]	0.79	0.432	1.234	0.04	[− 0.07, 0.15]	0.73	0.463	1.234
Threat	**0.32**	**[0.22, 0.42]**	**6.13**	** < 0.001**	**2.201**	0.13	[− 0.01, 0.27]	1.78	0.077	2.201

*Note.* Significant predictors are in bold. VIF = Variance Inflation Factor.

## Study 2

Study 1 showed that distrusting worldviews (conspiracy mentality, science populism and cynicism), and perceptions of threat are associated with approving attitudes toward harassment of scientists. Therefore, both worldviews and radicalization factors seem to play a role. However, not everyone with a given worldview or radicalization factors engages in extreme attitudes or behaviors. Additionally, these factors are more related to outlooks on society and the world (e.g., how it should look or how it is perceived), which does not account for individual (personality) factors. In Study 2, we also explored the role of personality, by including the most obvious candidate for extremely negative and violent attitudes, the dark triad: (subclinical) narcissism, Machiavellianism, and (subclinical) psychopathy.

### Methods

#### Participants

Similar to Study 1, we were interested in examining the pattern of correlations between multiple predictors and outcomes. Based on the power analysis (power analysis: *r* = 0.15; power = 80%, α = 0.05), we set to collect data from 343 participants. We recruited 359 participants from Prolific US who completed the study in return for £1.00 (approximately $1.26; note that Prolific is a UK platform that uses sterling pounds as a currency). After excluding 17 speeders (completion time at least twice as fast as the median) and 6 multivariate outliers on independent and dependent variables, we were left with *N* = 336 (155 males, M_age_ = 42.5). Regarding education, 2.1% indicated not having completed high/secondary school, 42.6% had completed high/secondary school, 4.5% were currently studying, 37.2% had an undergraduate degree and 13.7% had a graduate degree.

#### Materials

Questions about socio-demographics, worldviews, and risk factors of radicalization were identical to Study 1. All scales showed good or acceptable reliability: political ideology (correlation between two items *r* = 0.72), religiosity (α = 0.90), spirituality (correlation between two items *r* = 0.84), conspiracy mentality (α = 0.91), science populism (α = 0.83), science cynicism (α = 0.86), and attitudes towards scientist harassment (α = 0.88). As in Study 1, due to the very high correlation between collective and relative deprivation (*r* = 0.88), and symbolic and realistic threat (*r* = 0.84), we calculated one measure of relative deprivation (α = 0.95) and threat (α = 0.96).

Instead of the Voodoo Doll Task which showed low convergent validity (correlation of *r* = 0.11 with attitudes), we used two different behavioral measures. We asked participants to sign the petition against the harassment of scientists, providing them with the petition link (https://www.ipetitions.com/petition/stop-harassment-of-scientists; the petition outlook can also be found in Supplement), and asking them to fill out their first and last name, and email address. For the donation, we offered a bonus payment of £1.00 to participants, which they could distribute (between 0 and 100 pence) to themselves or donate to the Union of Concerned Scientists (www.ucsusa.org), which is a non-profit organization that also deals with the protection of scientists from harassment. Out of a total of £359 offered as a bonus, participants opted to donate 69.79£, which we transferred to the Union of Concerned Scientists.

##### Dark triad

To measure the three dark personality traits, we used the Dirty Dozen Scale^68^. Participants indicated how much they agreed with statements like “I tend to want others to admire me”, “I tend to want others to pay attention to me” (narcissism), “I tend to manipulate others to get my way”, “I have used deceit or lied to get my way” (Machiavellianism), and “I tend to lack remorse”, “I tend to be callous or insensitive” (psychopathy). Answers were given using a 5-point Likert scale ranging from *strongly disagree* (1) to *strongly agree* (5). Each of the three subscales included four items and showed good (narcissism: α = 0.84, Machiavellianism: α = 0.85) or acceptable reliability (psychopathy: α = 0.76).

### Results

Table 3 contains means, standard deviations, and intercorrelations of variables (other descriptives and assumption checks for linear regressions are available on OSF as supplemental information). Similarly to Study 1, attitudes toward harassment were related to all worldviews and radicalization factors (except, however, for religiosity and spirituality). Again, there was variation in correlation effect sizes, ranging between − 0.04 and 0.40 (for spirituality and threat perception respectively). Regarding the donation behavior, it followed the same pattern of relations with worldviews and risk factors as attitudes—it correlated with all of them, except religiosity and spirituality (note the negative sign as less donating suggested more negative behavior). Petition behavior was only correlated with political ideology and science cynicism (again, note the negative sign), the only two predictors associated with both behavioral variables. Surprisingly, behavioral measures showed no significant correlations with attitudes.

**Table 3:** Means, standard deviations and intercorrelations between variables.

	$M$	$SD$	1	2	3	4	5	6	7	8	9	10	11	12	13
1 Political ideology	5.15	2.18													
2 Religiosity	2.88	1.16	0.35***												
3 Spirituality	4.00	1.88	0.22***	0.81***											
4 Conspiracy Mentality	4.07	1.34	0.27***	0.22***	0.24***										
5 Science Populism	2.71	0.92	0.42***	0.33***	0.30***	0.45***									
6 Science Cynicism	2.66	0.95	0.53***	0.23***	0.17**	0.45***	0.65***								
7 Relative Deprivation	2.78	0.95	− 0.11	− 0.02	0.01	0.20***	0.24***	0.19***							
8 Threat	1.64	0.80	0.41***	0.29***	0.23***	0.43***	0.57***	0.61***	0.21***						
9 Psychopathy	1.95	0.74	− 0.06	− 0.28***	− 0.28***	0.02	0.00	0.12*	0.24***	0.04					
10 Machiavellianism	1.95	0.88	− 0.08	− 0.03	− 0.07	0.09	0.03	0.03	0.23***	0.06	0.53***				
11 Narcissism	2.18	0.90	− 0.03	0.09	0.05	0.11*	0.09	0.05	0.10	0.07	0.30***	0.51***			
12 Scientist Harassment	1.40	0.51	0.19***	0.03	− 0.04	0.21***	0.30***	0.39***	0.23***	0.40***	0.27***	0.22***	0.29***		
13 Donation	19.61	31.53	− 0.22***	0.00	0.01	− 0.21***	− 0.23***	− 0.28***	− 0.12*	− 0.16**	− 0.08	− 0.04	− 0.01	− 0.08	
14 Petition	0.18	0.39	− 0.16**	− 0.01	0.05	0.05	− 0.07	− 0.14**	0.06	− 0.10	0.07	0.18**	0.15**	0.00	0.17**

*p < 0.05, **p < 0.01, ***p < 0.001.

The dark triad was an extension of the first study. All three dark personality traits showed a positive relation to approving attitudes toward harassment. However, they either had no relations or were (somewhat unexpectedly) positively related to the two behavioral variables.

Like in Study 1, we assessed predictors’ relative importance for attitudes toward scientists’ harassment and donation behavior by conducting multiple linear regression analyses, while for the signing of the petition, we conducted logistic linear regression given that the dependent variable was binary (signed or not). We entered worldviews, radicalization risk factors, and dark triad personality variables as predictors (Table 4). For all three dependent variables, the overall models were significant. Larger science cynicism and threat perception were associated with more approving attitudes toward harassment. Dark triad also played a role, as psychopathy and narcissism were also associated with more harassment-approving attitudes. Regarding the behavioral variables, only political ideology predicted donation (right-wing individuals donated less), while none of the predictors was associated with signing the petition (even though the overall model was significant). Given that individual predictors in the regression model were not significant, we turned to zero-order correlations, which indicated that science cynicism was the only consistent predictor of both behavioral outcomes. As in Study 1, we ran the same regression analyses while including age, education, and gender as a control, showing the same pattern of results (fully reported in the supplement on OSF). Finally, as in Study 1, we did not run the pre-registered mediation analysis given weak effects on the behavioral variables.

**Table 4:** Worldviews and risk factors predicting attitudes toward scientist harassment, donation and petition behavior.

Model (DV and fit)	Scientist harassment					Donation					Petition				
	*F*(11,324) = 12.87, *p* < 0.001; *R*^2^ = 0.304 (adj *R*^2^ = 0.280)					*F*(11,324) = 3.98, *p* < 0.001;* R*^*2*^ = 0.120 (adjusted* R*^*2*^ = 0.090)					*Χ*^*2*^ (324) = 30.713, *p* = 0.001; McFadden* R*^*2*^ = 0.096; Cox & Snell *R*^2^ = 0.087				
Predictor	*β*	95%-CI	*t*	*p*	VIF	*β*	95%-CI	*t*	*p*	VIF	*β*	95%-CI	*z*	*p*	VIF
Political Ideology	0.02	[− 0.10, 0.14]	0.33	0.740	1.671	**− 0.16**	**[− 0.29, − 0.03]**	**− 2.35**	**0.019**	1.**671**	− 0.27	[− 0.65, 0.10]	− 1.43	0.152	1.513
Religiosity	0.03	[− 0.14, 0.19]	0.32	0.748	3.320	0.11	[− 0.08, 0.29]	1.12	0.264	3.316	− 0.18	[− 0.69, 0.34]	− 0.67	0.502	2.989
Spirituality	− 0.13	[− 0.29, 0.03]	− 1.61	0.107	3.026	0.01	[− 0.17, 0.19]	0.11	0.915	3.020	0.35	[− 0.15, 0.86]	1.38	0.167	2.866
Conspiracy Mentality	− 0.02	[− 0.13, 0.09]	− 0.41	0.681	1.418	− 0.11	[− 0.23, 0.01]	− 1.74	0.083	1.418	0.34	[− 0.02, 0.70]	1.84	0.065	1.387
Science Populism	0.03	[− 0.11, 0.16]	0.38	0.704	2.108	− 0.07	[− 0.22, 0.07]	− 0.99	0.323	2.113	− 0.04	[− 0.46, 0.39]	− 0.17	0.864	1.910
Science Cynicism	**0.19**	**[0.04, 0.33]**	**2.58**	**0.010**	**2.448**	− 0.16	[− 0.32, 0.00]	− 1.92	0.056	2.452	− 0.33	[− 0.81, 0.14]	− 1.36	0.173	2.196
Relative Deprivation	0.10	[− 0.01, 0.20]	1.85	0.066	1.253	− 0.08	[− 0.19, 0.04]	− 1.34	0.182	1.249	0.04	[− 0.29, 0.37]	0.24	0.808	1.282
Threat	**0.25**	**[0.13, 0.38]**	**4.01**	** < 0.001**	**1.851**	0.08	[− 0.06, 0.22]	1.07	0.286	1.848	− 0.25	[− 0.39, 0.38]	− 1.11	0.266	1.687
Psychopathy	**0.13**	**[0.01, 0.24]**	**2.11**	**0.036**	**1.671**	− 0.01	[− 0.14, 0.12]	− 0.16	0.870	1.666	− 0.00	[− 0.03, 0.73]	− 0.01	0.989	1.785
Machiavellianism	− 0.01	[− 0.13, 0.12]	− 0.10	0.917	1.794	− 0.02	[− 0.15, 0.12]	− 0.26	0.796	1.775	0.35	[− 0.11, 0.58]	1.81	0.071	1.854
Narcissism	**0.22**	**[0.11, 0.33]**	**3.99**	** < 0.001**	**1.408**	0.01	[− 0.11, 0.13]	0.21	0.831	1.399	0.23	[− 0.71, 0.18]	1.32	0.186	1.469

Significant predictors are in bold. VIF = Variance Inflation Factor.

## Discussion

In the past several years, perhaps especially during the COVID-19 pandemic, scientists were faced with different forms of harassment, from death threats to lynching attempts^2,37^. But who are the individuals that engage in such behavior toward scientists? The present research probed worldviews (religiosity, spirituality, political ideology, conspiracy mentality, science populism, and cynicism), risk factors of radicalization (relative deprivation and threat), and personality (dark triad) as potential drivers of scientist harassment. Across two studies, we found that distrusting worldviews (conspiracy mentality, science populism and science cynicism), political ideology, and perception of threat are associated with more approving attitudes toward scientists’ harassment. Assessing their relative importance, we found that science cynicism and perception of threat are consistently associated with attitudes. Additionally, Study 2 illuminated the role of dark personality traits, as psychopathy and narcissism also contributed to approving attitudes toward harassment. Explaining actual behavior turned out to be more challenging – only science cynicism was associated with negative behavior consistently (for all three behaviors across two studies). However, assessing the relative importance of the other predictors in behaviors did not yield conclusive results: while the overall regression models explained variance, there was no consistent pattern of the role of individual predictors.

In sum, viewing scientists as incompetent and corrupt (science cynicism) drives scientist harassment (both attitudes and behavior). Additionally, the perception of scientists as a threat, narcissism, and psychopathy contributed to approving scientists’ harassment. Noteworthy, although our studies are correlational, we believe that the included predictors (more general worldviews formed earlier) indeed cause the more specific attitudes and behaviors regarding scientists, which is in line with much work in the area (e.g.,^3^).

### Who approves of scientists’ harassment?

#### The role of worldviews

Previous research has shown that worldviews such as political or religious ideology are related to science skepticism or mistrust of science (e.g.,^3,25^). While some of the worldviews also relate to different forms of violence (e.g., right-wing ideology^39^, conspiracy mentality^42^), it has been virtually unknown whether any of the worldviews contribute to the harassment of scientists. We found that most of the worldviews identified as driving negative attitudes toward science and scientists^13,25^ also apply to harassment: right-wing ideology and distrusting worldviews (conspiracy mentality, science populism, and cynicism) were consistently associated with approving harassment of scientists. However, for religiosity and spirituality, this was only the case in Study 1 but not in Study 2. Given that the credo of most religions is not to harm (e.g., Exodus 20:13) or that religion can even play a protective factor against violent behavior (e.g., Salas-right et al.^69^), it is not surprising that religiosity is not necessarily related to more approving attitudes of scientists’ harassment even though they generally relate to more negative attitudes toward science and scientists.

Assessing relative importance showed that science cynicism emerged as the most prominent predictor of attitudes and behaviors. What makes cynicism a main driver of scientist harassment? As with conspiracy mentality and populism, at the core of cynicism lies fundamental distrust predominantly aimed at elites and the establishment (which scientists are seen as a part of). Cynical individuals doubt scientists’ motives, competence, and morality, thus creating a fertile ground for scientists’ harassment. However, we note that the measures of populism and cynicism we used specifically referred to scientists, unlike conspiracy mentality (which contained generalized statements). Therefore, it is difficult (and possibly premature) to tease apart which of the three distrusting worldviews plays a more important role, especially since novel research indicates that they are more similar than previously thought^35,36^. Indeed, we found a high intercorrelation between the three distrusting worldviews (*r*s ranging between 0.45 and 0.68 across two studies), suggesting their conceptual overlap. Overall, it is interesting that trust or distrust—which hitherto has been mostly studied as an outcome in science attitudes research (e.g.,^7,13,70^—has its own outcomes, dangerous in its own regard.

#### Harassment of scientists as a form of radicalization: risk factors

Building upon radicalization frameworks^45–48^ which suggested that risk factors of relative deprivation and threat are one of the crucial drivers of radicalization, we used this approach to understand the harassment of scientists. Both risk factors were associated with more approval of scientists’ harassment, although the perception of threat consistently emerged as more prominent when we assessed their relative importance.

Even though it is understandable that areas in which scientists work (e.g., virology, climate change, nuclear energy) can be perceived as threatening, why would someone perceive scientists as such? First, it is true that although scientists work to prevent certain threats (e.g., COVID-19, climate crisis), some of their work also produces new threats (e.g., nuclear weapons and nuclear energy; Ho et al.^71^). Secondly, some research, such as the development of novel technologies (e.g., bioengineering, AI), can also be perceived as threatening (e.g.,^51,72^). Lastly, as mentioned earlier, certain individuals could perceive the very solutions that scientists propose as threatening, restricting their financial and personal freedom^3,50^. These include propositions of structural changes in the economy, lifestyle changes to combat climate change, or mandatory mask-wearing, and physical distancing in COVID-19. All this also fits within negative stereotypes of “mad” or dangerous scientists, who have the capability and intention to cause harm to the world (e.g.,^73,74^). Overall, the current approach suggests that investigating scientists’ harassment as a radicalization phenomenon is promising for understanding why people engage in such behavior. Future research could also look into other factors of radicalization: for example, blaming the death of a family member on scientists (e.g., due to the perceived inability to contain COVID-19) could be a trigger factor.

#### Personality factors

Previous research found that dark triad personality is related to aggression and violence^53,56,58,59^. The current results are in line with these findings as dark triad personality was associated with approving attitudes toward harassment of scientists. Although we focused on the dark triad as the most obvious candidate, it would also be beneficial to see whether basic personality traits (e.g., the big five) might play a role. It would particularly be interesting to see the role of (low) agreeableness, which has been suggested to lie at the center of the dark triad personality^75^, but also see^76^. In this vein, reactance (tendency to react against perceived restrictions was found to be one of the strongest predictors of rejecting scientific evidence (e.g., vaccination^29^). Therefore, this basic disagreement as a reaction to any restriction could play an important role in negative attitudes toward scientists more generally, and consequently drive individuals to harm scientists.

### Attitudes toward scientists’ harassment and behavior

Although attitudes and behavior of scientists’ harassment had similar predictors (e.g., conspiracy mentality, science cynicism, deprivation and threat in Study 1, political ideology and science cynicism in Study 2), there was a noticeable issue. Attitudes and behaviors correlated slightly in Study 1 (*r* = 0.11) or not at all in Study 2 (*r* = 0.08 and 0.00), while the correlation between the two behaviors in Study 2 was also not high (*r* = 0.17). There are different potential explanations for these low/absent correlations. First, their content was different, as they related to harassment (attitude measure), physical activity (clicking), donation, and petition, which all do not necessarily go hand in hand with violence. Second, it is well-known that attitudes are only one of the drivers of behavior (e.g.,^77^). Next, the relationship between attitudes and behavior is stronger when they correspond in terms of generality versus specificity. Given that the behavioral measures were more specific than the attitudes scale, this could have caused lower correlations. Finally, measuring violent (i.e., anti-social) behaviors is likely influenced by social desirability (e.g.,^78^). This remains a significant limitation of the study, and future research on harassment of scientists should dedicate attention to how dependent variables are operationalized (e.g., multiple measures of attitudes), and how issues such as social desirability or generality and specificity of dependent variables can be overcome.

### Limitations and future research

The previously discussed point also relates to the possibility of selection bias given that individuals with strong harassment intentions toward scientists might have avoided participating. We acknowledge this limitation while noting that we named the study “Perceptions of Scientists” to mitigate this concern somewhat. Apart from this, concerns could be raised about the data quality in the present studies (e.g., no attention checks, only English language survey in Study 1 which includes non-native participants). However, we believe that data quality is high given that we did exclude speeders and outliers, and measures had high reliabilities while correlations between different worldviews were in the right direction (e.g., religiosity and spirituality were highly correlated). Secondly, there are some discrepancies in the results between the two studies (e.g., conspiracy mentality and science populism were significant predictors of attitudes in the regression in Study 1 but not in Study 2). However, we believe this is likely due to random sample variability, which can lead to fluctuations in the significance of regression coefficients. Therefore, we focused on the pattern of correlations when interpreting the results (both conspiracy mentality and science populism were positively correlated in both studies). Future research should further investigate which worldviews are more important and stable predictors. Another limitation includes our exclusive focus on perceptions of “scientists”, although increasing research shows that perceptions of scientists vary across disciplines^79,80^. Our studies are first in the area to address harassment of scientists, which is why we started by examining harassment of scientists in general. Future research should see whether and how our findings vary across perceptions of different scientific occupations and disciplines. It is very likely that some scientific occupations (e.g., those working on publicly contentious topics) will draw more negative perceptions than others.

## Conclusion

Even though scientists usually work on solutions to societal problems, they simultaneously experience harassment that can be as extreme as death threats and physical attacks. Drawing on the literature on science attitudes, radicalization, and personality, the present research is, to the best of our knowledge, the first to identify factors associated with scientist harassment. We discovered that harassment of scientists likely stems from feelings of cynicism (distrust) and threat, as well as from dark personality traits. Thus, the current research helps understand not only which individuals are more likely to harass scientists, but also what can be potentially done to curb it. Highlighting reasons why scientists should be trusted—and not threatened by—seems like the most promising way to counter such extremely negative behavior.

## Data Availability

Data is available at the Open Science Framework: https://osf.io/7rzce/?view_only=957180de7bcc4d0c88dedc2219e52816.
